# The Relationship between *VEGFA* and *TGFB1* Polymorphisms and Target Lesion Revascularization after Elective Percutaneous Coronary Intervention

**DOI:** 10.1155/2017/8165219

**Published:** 2017-07-24

**Authors:** Tadeusz Osadnik, Andrzej Lekston, Kamil Bujak, Joanna Katarzyna Strzelczyk, Lech Poloński, Mariusz Gąsior

**Affiliations:** ^1^2nd Department of Cardiology and Angiology, Silesian Center for Heart Disease, Zabrze, Poland; ^2^3rd Department of Cardiology, School of Medicine with the Division of Dentistry in Zabrze, Medical University of Silesia in Katowice, Silesian Center for Heart Disease, Zabrze, Poland; ^3^Department of Medical and Molecular Biology, School of Medicine with the Division of Dentistry in Zabrze, Medical University of Silesia, Katowice, Poland

## Abstract

**Background and Aim:**

The specific association between genetic variation and in-stent restenosis is still only partly understood. The aim of this study is to analyze the relationship between functional polymorphisms in the genes encoding vascular endothelial growth factor A (VEGF-A; rs699947) and transforming growth factor beta 1 (TGF-*β*1; rs1800470) and target lesion revascularization (TLR) risk.

**Methods:**

A total of 676 patients (805 lesions) with stable coronary artery disease (SCAD) who received elective percutaneous coronary intervention (PCI) with at least one bare-metal stent implantation were included. The primary study endpoint was TLR at a 4-year follow-up.

**Results:**

The TLR rate was higher in patients with the *VEGFA* A/A genotype (15.4%) than in patients with the *VEGFA* A/C (7.9%) and C/C (8.9%) genotypes (*p* = 0.009). The *VEGFA* A/A genotype, after adjustment for clinical and procedural covariates, remained significantly and independently associated with the TLR (hazard ratio—2.09 [95% confidence interval 1.32–3.33, *p* = 0.0017]). However, we found no association between TLR and the *TGFB1* genotype.

**Conclusion:**

The *VEGFA* A/A genotype is significantly and independently associated with TLR risk in Polish SCAD patients who received elective PCI with bare-metal stent implantation.

## 1. Background

In-stent restenosis (ISR) is a major limitation of percutaneous coronary intervention (PCI). To date, the etiology and genetic basis of this phenomenon are only partly understood. A wide array of inflammatory cytokines, growth factors, and mitogens as well as abnormal regional wall shear stress leads to intimal hyperplasia [1–6]. The authors hypothesize that polymorphisms in specific genes generate individual differences in the vascular wound healing process in response to wall injury after stent implantation.

Experimental studies have proven the important role of vascular endothelial growth factor A (VEGF-A) and transforming growth factor beta 1 (TGF-*β*1) in the formation of neointima and ISR development [7]. Nonetheless, the relationship between polymorphisms in genes encoding these growth factors and target lesion revascularization (TLR) risk has not been analyzed before. Therefore, we aimed to determine whether the polymorphisms in *VEGFA* (rs699947) and *TGFB1* (rs1800470) are associated with TLR in a prospective, population-based cohort of Polish patients who underwent PCI with bare-metal stent (BMS) implantation. We chose rs699947 and rs1800470 as previous studies confirmed that they are functional polymorphisms and affect both gene expression and VEGF-A and TGF-*β*1 serum levels [8, 9]. Moreover, we previously reported that rs699947 and rs1800470 polymorphisms in the genes encoding VEGF-A and TGF-*β*1, respectively, are associated with late lumen loss (LLL) in patients with stable coronary artery disease (CAD) who received elective PCI with BMS implantation [10]. Additionally, these polymorphisms have been studied in the context of other cardiovascular disorders and have been proven to affect, inter alia, the angiographic severity of CAD [11, 12] and the risk of myocardial infarction [13, 14].

## 2. Methods

### 2.1. Patient Population

We enrolled 676 Caucasian patients (805 lesions) with stable CAD who underwent elective PCI between January 2007 and December 2012 with the implantation of at least one BMS. The patients were not related to each other. The primary study endpoint was TLR at a 4-year follow-up, which was defined as either repeat percutaneous revascularization for a lesion anywhere within the stent, within 5 mm of the previously implanted stent, or the need for surgical revascularization of the stented vessel due to ISR. During index hospitalization, demographic and clinical data as well as periprocedural variables were recorded, including stent localization and diameter, the total stent length per lesion, and the number of stents implanted. Follow-up data on TLR were collected during subsequent hospitalizations. The survival information was based on a National Health Fund insurance status because a National Health Fund insurance policy is obligatory for all Polish citizens [15, 16].

### 2.2. Genotyping

DNA was extracted from blood samples using the GeneMATRIX Quick Blood DNA Purification Kit (EURX, Poland) according to the manufacturer's instructions. Similar to our previously used methods [10, 17], we identified single-nucleotide polymorphisms (SNPs) in the *TGFB1* and *VEGFA* genes using TaqMan genotyping assays on the 7300 Real-Time PCR System and the SDS 1.4 Allelic Discrimination software (Applied Biosystems, USA). Samples that were initially identified as homozygous and heterozygous were sequenced, and after genotype confirmation, they were used as positive controls. DNase-, RNase-, and protease-free water (Qiagen, Germany) was used as the negative control. For quality control, 10% of the samples were randomly repeated and showed complete agreement.

### 2.3. Statistical Analysis

Continuous variables are reported as the mean ± standard deviation. Categorical variables are presented as percentages. The chi-square test was used to determine whether the analyzed genotypes agreed with the Hardy-Weinberg equilibrium. Minor allele frequencies were calculated and reported. The Kaplan-Meier method was used to study the cumulative incidence of TLR overtime, whereas the log-rank test was applied to evaluate differences between patients with different *VEGFA* and *TGFB1* genotypes using the dominant model (homozygous major versus heterozygous and homozygous minor) and codominant model setting (homozygous major versus heterozygous versus homozygous minor). Patients who died before TLR occurred were censored at the time of death. To adjust for baseline clinical and periprocedural variables after positive evaluation of the proportional hazards assumption, Cox regression analysis was performed using TLR as a dependent variable. Prior to Cox regression analysis, the missing values were replaced using a state-of-the-art statistical method for mixed-type data imputation, the MissForest algorithm, to minimize information loss and the necessity to exclude entire cases due to single missing values. The MissForest algorithm is considered superior to other multiple imputation methods such as multivariable imputation by chained equations (MICE) [18]. Every variable, including the outcome variable, was included in the multiple imputation algorithm. To visualize the impact of the *VEGFA* and *TGFB1* genotypes on TLR, the adjusted Kaplan-Meier curves were plotted using the inverse probability weight method and compared with the log-rank test for adjusted curves. To account for multiple comparisons resulting from multiple tests of *VEGFA* and *TGFB1* SNPs using the dominant and codominant models, a Bonferroni-corrected *p* value = 0.05/4 = 0.0125 was considered statistically significant. Statistical analyses were entirely performed using R software and freely available statistical packages [19–21].

The study conformed to the Declaration of Helsinki and was approved by the Ethics Committee of the Silesian Medical Chamber in Katowice, Poland.

## 3. Results

**Figure 1:**
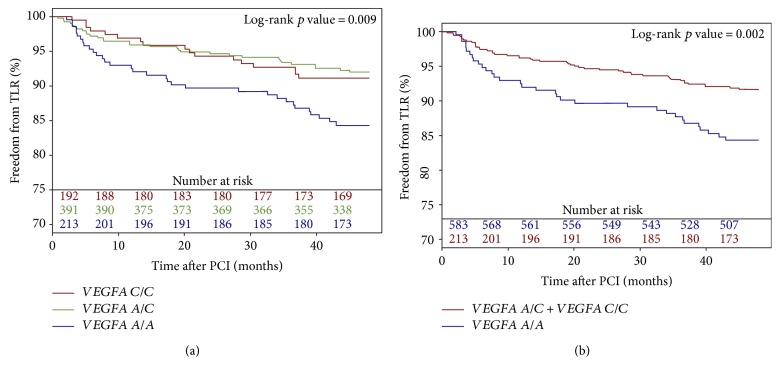
Freedom from TLR according to the *VEGFA* polymorphism genotypes using the codominant (a) and dominant model (b).

**Figure 2:**
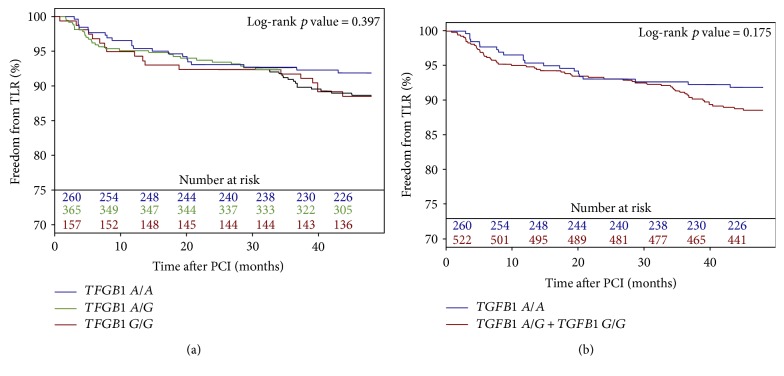
Freedom from TLR according to *TGFB1* polymorphism genotypes using the codominant (a) and dominant model (b).

**Figure 3:**
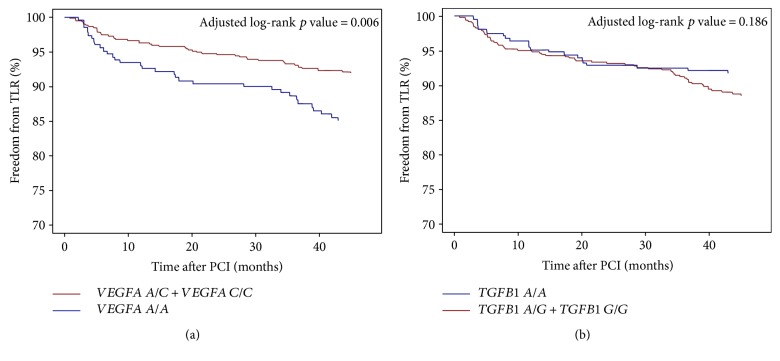
Freedom from TLR according to the *VEGFA* (a) and *TGFB1* (b) polymorphism genotypes adjusted for clinical and periprocedural covariates.

**Figure 4:**
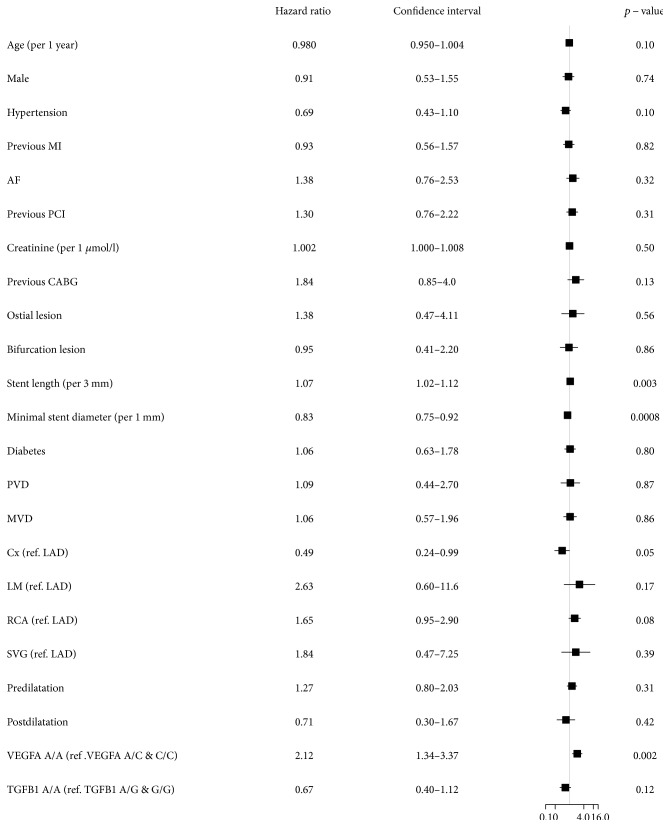
Multivariate analysis of the impact of variables associated with clinical and periprocedural characteristics on 4-year TLR. TLR: target lesion revascularization; SCAD: stable coronary artery disease; PCI: percutaneous coronary intervention; BMS: bare-metal stent; MI: myocardial infarction; AF: atrial fibrillation; CABG: coronary artery bypass grafting; PVD: peripheral vascular disease; MVD: multivessel coronary disease; Cx: circumflex branch; LAD: left anterior descending; LM: left main; RCA: right coronary artery; SVG: saphenous vein graft.

Baseline clinical and periprocedural characteristics are presented in Table 1. The *VEGFA* and *TGFB1* genotype distribution agreed with the Hardy-Weinberg equilibrium, and minor allele frequencies were similar to those reported for European populations (Table 2). Genotypes of *TGFB1* and *VEGFA* were successfully established for 663 (98.1%) and 675 (99.9%) patients, respectively. The 48-month follow-up was available for 670 (99.1%) patients. During the follow-up period, 25 (3.7%) patients died. The TLR rate was higher in patients with the *VEGFA* A/A (15.4%) genotype than in patients with the *VEGFA* A/C (7.9%) and C/C (8.9%) genotypes using the codominant (Figure 1(a)) and dominant models (Figure 1(b)) (*p* = 0.009 and *p* = 0.002, resp.). There were, however, no differences in TLR frequency for different *TGFB1* genotypes (A/A—8.4%, A/G—11.2%, and G/G—11.5%) using the codominant (Figure 2(a)) and dominant (Figure 2(b)) models (*p* = 0.397 and *p* = 0.175, resp.). The *VEGFA* A/A genotype, after adjustment for clinical and periprocedural covariates, remained significantly and independently associated with TLR (adjusted log-rank *p* = 0.006, hazard ratio (HR)—2.09 [95% confidence interval (CI) 1.32–3.33, *p* = 0.0017]; Figures 3(a) and 4()), whereas no association was observed for the *TGFB1* A/A genotype (ref. A/G and G/G; adjusted log-rank *p* = 0.186, HR—0.67 [95% CI 0.40–1.12, *p* = 0.12]; Figures 3(b) and 4()). Other factors associated with TLR were minimal stent diameter (HR (per 1 mm increase)—0.43 [95% CI 0.26–0.7, *p* = 0.0008]), stent length (HR (per 3 mm increase)—1.08 [95% CI 1.03–1.13, *p* = 0.003]), and lesions localized in the circumflex branch of the left coronary artery (reference LAD) (HR—0.49 [95% CI 0.24–0.99, *p* = 0.04]) (Figure 4()).

## 4. Discussion

Atherosclerosis is considered a multifactorial disease influenced by environmental and genetic factors. Furthermore, the pathophysiological mechanisms of restenosis have also not yet been fully explained. Coronary angioplasty injures the arterial wall, leading to parietal thrombus formation and a local inflammatory response, which is considered the main driver of vascular smooth muscle cell (VSMC) proliferation and neointima formation [22]. VSMC proliferation is stimulated by the cytokines released from monocytes/macrophages [3], and studies have shown that one week after PCI, the neointima contains 60% VSMCs and 30% neutrophils and monocytes. In the subsequent weeks, the number of mononuclear cells decreases, which is accompanied by a significant increase in the VMSC cell percentage. Four weeks after stent implantation, over 90% of the cells that form the neointima are VMSCs [23]. Therefore, many genetic studies on restenosis have examined genes encoding inflammation-related proteins, particularly polymorphisms in the genes for interleukin-1, interleukin-10, interleukin-1 receptor antagonist, matrix metalloproteinases, and C-reactive protein [24, 25]. Mediators of inflammation produced by monocytes and macrophages stimulate the release of interleukins, TGF-*β*1, and other growth factors, specifically, VEGF-A. These proteins modulate the proliferative activity of endothelial cells and VSMCs and affect adhesion molecule expression [26, 27].

### 4.1. rs1800470 Polymorphism (*TGFB1*)

TGF-*β*1 is a cytokine with a complex mechanism of action. TGF-*β*1 mainly stimulates TGF-*β* receptor type II (TGFBR2), which recruits TGF-*β* receptor type I (TGFBR1) to form a complex (TGF-*β*1 + TGFBR2 + TGFBR1) that activates the Smad pathway. Throughout this pathway, TGF-*β*1 exhibits antiproliferative [28] and anti-inflammatory effects [29], accelerates cell differentiation [30], and promotes extracellular matrix synthesis [31]. TGF-*β*1 released locally by arterial wall fibroblasts sends paracrine signals to the VSMCs and the macrophages migrating toward the injured region of the stented arterial wall. The platelets that participate in parietal thrombus formation release large amounts of TGF-*β*1 [32]; furthermore, serum TGF-*β*1 levels might be genetically determined. According to some studies, the presence of allele C correlates with higher TGF-*β*1 concentrations [23–34]. Other researchers have observed increased TGF-*β*1 levels in T/T genotype carriers [35]. TGF-*β*1 inhibits proliferation in G1 phase, although at levels higher than 1-2 fg per cell, it might promote smooth muscle cell, fibroblast, and chondrocyte proliferation [36]. There is no linear correlation between TGF-*β*1 expression and its effect on the cells in the restenosis process. Studies performed at our center have not shown a relationship between serum TGF-*β*1 concentration and history of restenosis, including recurrent restenosis and the first restenosis [37, 38]. Recently, Chung et al. reported that blocking TGF-*β*1 by intravascular local gene delivery does not reduce neointima formation but enhances the inflammatory response in a pig model of restenosis, which potentially aggravates lesion progression [39]. Previous studies indicated that the rs1800470 genotype is associated with the risk of developing cardiovascular diseases and their complications, inter alia, cerebral infarction [40], silent myocardial ischemia in diabetic patients [41], and CAD complications [13]. Yang et al. investigated the relationship between the rs1800470 polymorphism and angiographic severity of CAD in Chinese population. They found that allele T is associated with higher CAD burden assessed using the Gensini Score [11]. On the other hand, a study conducted at our center did not confirm these findings in the population of Polish patients [17]. *TGFB1* polymorphism (rs1800470) has been also studied in the context of ISR. Fragoso et al. reported, for the first time, that rs1800470 polymorphism could be involved in the risk of developing ISR in the Mestizo population undergoing PCI with drug-eluting stent or BMS implantation [42]. In our previous study, we showed that the *TGFB1* polymorphism (rs1800470) allele T is associated with decreased neointima formation in patients with CAD receiving BMS [10]. Any discrepancy with our previous study can be explained by the use of different inclusion criteria and the different endpoints of both studies.

### 4.2. rs699947 Polymorphism (*VEGFA*)

VEGF-A is a potent and highly specific endothelial cell mitogen that regulates endothelial integrity [43–45], although in the literature, there is still a debate regarding whether VEGF-A is a proatherosclerotic or antiatherosclerotic factor [12]. rs699947 is a functional polymorphism associated with VEGF-A levels. In particular, Shahbazi et al. reported that the C/C genotype is associated with higher VEGF-A synthesis than the A/A genotype [46]. Howell et al. genotyped 941 patients with CAD for the rs699947 polymorphism, and the A/A genotype frequency increased stepwise with the number of diseased coronary arteries using the C/C genotype as the reference. Therefore, the A/A genotype is a risk factor for atherosclerosis, and the C/C genotype is protective [12]. Our previous study evaluating the Gensini Score as a marker of atherosclerotic burden relative to SNPs revealed that the A/A genotype was more frequently observed than the C/C genotype in patients with the highest Gensini Score [17]. Results of the meta-analysis of seven case-control studies indicated that rs699947 may be associated with the risk of CAD development, and A allele carriers have higher CAD susceptibility in comparison with the C allele carriers [47]. Furthermore, the rs699947 *VEGFA* polymorphism is associated with collateral circulation in CAD patients [48] and myocardial infarction risk in patients with rheumatoid arthritis [14] as well as may affect the antihypertensive responses to enalapril [49]. In our previous analysis, we showed that the A/A genotype is a risk factor for increased neointima formation, whereas the C/C genotype was protective [10]. On the other hand, we did not find any relationship between *VEGFA* rs699947 and the risk of binary ISR [10]. Moreover, Bagyura et al. who analyzed the relationship between *VEGFA* polymorphisms and the risk of ISR in patients who underwent PCI with BMS implantation reported that rs699947 polymorphism is associated with neither the risk of diffuse nor focal ISR [50]. Our current analysis shows for the first time that the rs699947 A/A genotype is associated with a higher TLR risk. Similar to the results of the analysis of *TGFB1* polymorphism, the discrepancies with previous studies regarding the role of *VEGFA* in ISR development could be associated with different inclusion criteria and different study endpoints.

### 4.3. Conclusions

In summary, we report for the first time that the rs699947 polymorphism in the *VEGFA* gene is associated with TLR in patients with stable CAD receiving PCI with stent implantation. This study suggests that a genetic polymorphism in *VEGFA* might be applicable to risk stratification for TLR. More detailed genetic studies in different ethnic populations are needed to further evaluate the association between *VEGFA* polymorphisms and ISR.

## Figures and Tables

**Table 1:** Baseline clinical and procedural characteristics.

*Clinical characteristics (n* = 676* patients)*		
Age (years)		63.4 ± 9.3
Female		194 (28.7)
Hypertension		467 (69.1)
Diabetes mellitus		181 (26.8)
Previous myocardial infarction		369 (54.6)
Atrial fibrillation		89 (13.1)
Previous PCI		277 (41)
Previous CABG		66 (9.8)
Creatinine (*μ*mol/l)		84.3 ± 34.1

*Procedural characteristics (n* = 805* lesions)*		

Vessel treated	LM	15 (1.9)
	LAD	220 (27.3)
	Cx	257 (31.9)
	RCA	300 (37.3)
	SVG	13 (1.6)
Ostial lesion		19 (2.4)
Bifurcation lesion		75 (9.3)
Number of stents implanted per lesion		1.1 ± 0.34
Total stent length per lesion (mm)		19.5 ± 9.4
Minimal stent diameter (mm)		3.01 ± 0.54
Predilatation		412 (51.2)
Postdilatation		72 (8.9)

Continuous variables are presented as the mean ± standard deviation. Categorical variables are presented as number of patients/lesions (percentages). PCI: percutaneous coronary intervention; CABG: coronary artery bypass grafting; LM: left main; LAD: left anterior descending; Cx: circumflex branch; RCA: right coronary artery; SVG: saphenous vein graft.

**Table 2:** Distribution of *TGFB1* and *VEGFA* polymorphism genotypes in the analyzed patient cohort (*n* = 676).

Gene/polymorphism	Homozygous major	Heterozygous	Homozygous minor	HWE *p* value	MAF	MAF EU populatio*n* [51]
	A/A	A/G	G/G			
*TGFB1* (rs1800470)	224 (33.8%)	310 (46.8%)	129 (19.5%)	0.27	42.8	38
	A/A	A/C	C/C			
*VEGFA* (rs699947)	186 (27.6%)	322 (47.7%)	167 (24.7%)	0.25	48.6	50

Genotypes of *TGFB1* and *VEGFA* were successfully established for 663 (98.1%) and 675 (99.9%) patients, respectively. MAF: minor allele frequency; EU: European Union; HWE: Hardy-Weinberg equilibrium.
